# Understanding heat patterns produced by vehicular flows in urban areas

**DOI:** 10.1038/s41598-017-15869-6

**Published:** 2017-11-24

**Authors:** Rui Zhu, Man Sing Wong, Éric Guilbert, Pak-Wai Chan

**Affiliations:** 1 Department of Land Surveying and Geo-Informatics, The Hong Kong Polytechnic University, 181 Chatham Road South, Kowloon, Hong Kong; 2 Department of Geomatics Sciences, Laval University, 1055 Avenue du séminaire, Local 1327, Québec (Québec), G1V 0A6 Canada; 3 Hong Kong Observatory, 13A Nathan Road, Kowloon, Hong Kong

## Abstract

Vehicular traffic has strong implication in the severity and degree of Urban Heat Island (UHI) effect in a city. It is crucial to map and monitor the spatio-temporal heat patterns from vehicular traffic in a city. Data observed from traffic counting stations are readily available for mapping the traffic-related heat across the stations. However, macroscopic models utilizing traffic counting data to estimate dynamic directional vehicular flows are rarely established. Our work proposes a simple and robust cell-transmission-model to simulate all the possible cell-based origin-destination trajectories of vehicular flows over time, based on the traffic counting stations. Result shows that the heat patterns have notable daily and weekly periodical circulation/pattern, and volumes of heat vary significantly in different grid cells. The findings suggest that vehicular flows in some places are the dominating influential factor that make the UHI phenomenon more remarkable.

## Introduction

Urban Heat Island (UHI) is an environmental phenomenon characterized by temperatures in urban areas being significantly higher than in surrounding rural areas. It is one of the major environmental issues caused by urbanization that generates more heat and adverse effects in local climate^1,2^, increasingly receiving public concern during the recent decades. UHI causes various adverse impacts to society in terms of health risk^3–5^, public security^6–8^, and energy consumption^9,10^. In addition to estimating the magnitude of the UHI intensity, literature has been explicitly studied on their formation mechanism. They gradually reached a consensus that UHI is caused by (i) loss of greenery area over urbanization^11–14^; (ii) buildings blocking ventilation corridors and accumulating heat^15,16^; (iii) construction materials with low specific heat capacities absorbing solar radiations or reflecting solar radiations in densely built up areas^17^; and (iv) increase of vehicles and growing electricity consumption producing more anthropogenic heat^18–20^. With regard to anthropogenic heat, vehicles can generate large amount of heat, and heat dispersion can be slowed down because of dense road networks and clusters of high-rise buildings^21,22^. For example, the highest UHI intensity can be observed in the Kowloon peninsula of Hong Kong along major roads and road intersections, with a significant number of vehicles passing through every day^23^. Thus, vehicular traffic should be considered as one of the major causes that increase the severity of UHI especially in mega cities such as Hong Kong. The objective of this study is to develop a quantitative approach that can investigate the influence of vehicle movements on UHI.

Understanding the influence of vehicular flow on UHI requires an accurate estimation of the time-dependent traffic flows, i.e., the number of directional moving vehicles passing through a road network at a given time period. Traffic flow estimation in literature is mainly divided into two categories: microscopic traffic modeling which estimates the behavior of each individual vehicle^24,25^ and macroscopic traffic modeling which describes the characteristics of traffic flows using aggregated parameters such as density and average speed^26,27^. Microscopic models normally collect sporadic data with spatial information (e.g. GPS locations) to construct the trajectory of each vehicle, which is depicted as a time-series of vehicle locations^28^. Hence, the models can estimate heterogeneous traffic flows appropriately since origin–destination (OD) matrices can be derived explicitly. This approach is effective to reveal spatio-temporal traffic flow patterns but fails to provide reliable quantitative information of the vehicular traffic, since recording real-time location-based information of every vehicle is still a challenge. A possible solution is through supersampling to extrapolate a system^29^, which requires a complex maximum entropy model. In contrast, macroscopic models usually utilize data collected from traffic sensors (e.g. traffic counting stations), and can overcome this problem because data are available for aggregation with steady and frequent updating. Therefore, macroscopic models usually have fewer variables and need fewer properties^26,27^, which can simplify the computation of heat flux accumulation with higher reliability. A recent study incorporated vehicle-driver behaviors into the macroscopic models^30^, in which the behaviors were derived from microscopic traffic flows.

For macroscopic models, dynamic traffic assignment (DTA) can be used to estimate traffic flow patterns on the road network. It is because DTA is formed by a principle of travel option which can determine (i) departure times, (ii) origins and destinations, (iii) travel routes of the vehicles, and (iv) a traffic flow module, that it can trigger the propagation of traffic flows over time^31^. To obtain better inherent consistency of dynamic routing behavior, substantial studies have used a time-series of traffic counts to develop time-dependent origin-destination (TDOD) estimation^32–36^. Specifically, several bi-level optimization models, which contain an upper-level problem to represent trip matrix of the OD demands and a lower-level problem to assign dynamic traffic flows, have been proposed by assuming the OD demand is either stochastic^32–36^ or deterministic^37–41^. Even though the stochastic approach does not require prior knowledge to adjust functional relations between the parameters, the searching capability is relatively weak and is much time consuming. In contrast, the deterministic algorithms are much more effective to solve the upper-level problem since explicit OD demands can be determined by executing a DTA simulator only several times to aggregate traffic volumes measured from traffic stations^42^.

A cell-transmission-model (CTM), in which vehicles travel through a set of grid cells, has gained wide attentions because of its simplicity to apply the Lighthill-Whitham-Richards (LWR) model to describe traffic dynamics as density-speed related flows^43,44^. Compared with LWR models, better results were achieved by employing higher-order models^45,46^. However, these models rely on complex equations and algorithms. To simplify the model and to adapt this model to a big-data computational module at the best convenience, this study will still use a LWR model to investigate a series of parameters for refining the results.

A recent study incorporated a TDOD demand estimation with a CTM to simulate dynamic traffic flows on the road network^44^. In the study, the OD demand estimation was transformed to solve the excess-demand DTA problem in order to optimize three equilibrium conditions (i.e., minimized routing costs, minimized traffic-count matching errors, and maximized OD demand entropies). In comparison, our work develops a similar but simpler and more robust bi-level TDOD demand estimation model that can (i) establish travel behaviors and TDOD trip matrix before iterative computations; (ii) focus on the vehicular flow assignment in each iterative computation; and (iii) achieve the model refinement through systematic post tests instead of instant optimization. In addition, this framework can model weekly dynamic traffic flows and can estimate accumulative heat flux generated from vehicles from weekday to weekend in Hong Kong. In this study, moving behavior of vehicles is firstly modeled by considering the turning probabilities of each vehicle at road intersections. Road networks are then discretized by a series of spatial continuity of homogeneous cells, and discretized networks are generalized as directional dummy paths in group of each cell such that the accessibility of vehicles from each traffic counting station to the cell boundaries and the accessibility from each boundary to its adjacent boundaries can be derived. Further, traffic counting stations are viewed as explicit origins and destinations, and the cells where parking lots and roadside parking areas are determined, so that deterministic TDOD demands can be constructed based on a time-series analysis. The above foundation can significantly reduce the computational load of estimating dynamic traffic flows in each iteration. Thus, trajectories of station-to-boundary and boundary-to-boundary traffic flows can be simulated by a new CTM in-cooperation with the elimination of duplicated counting of vehicles, and for estimation of the traveling speed. To validate the effectiveness of the proposed model, boundary-to-station vehicular flows with the quantitative information are simulated simultaneously for each time period so that it can be used to compare with the data observed from the stations. Lastly, heat flux produced by vehicles can be accumulated in each cell for each constant time period, which is hence used for the correlation analysis with air temperatures observed by automatic weather stations.

Road network used in this study was obtained from OpenStreetMap^47^, and the traffic flow was simulated based on traffic counting data for the entire year of 2015, which were provided by the Transport Department of Hong Kong (TDHK). Source data for different types and numbers of vehicles and their fuel statistics were obtained from TDHK^48^ and Electrical and Mechanical Services Department of Hong Kong^49^ for accumulating heat flux. Hourly air temperatures for the same year acquired from the Hong Kong Observatory were used for the model validation.

## Results

### Moving probability in the shortest path

This work assumes that all the vehicles follow the shortest paths (SPs) from their origins *o* to the destinations *d*. For example, urban population gives higher promises to drive on major roads or highways from their homes to workplaces since these paths are easily accessible and time effective. By assigning higher weights to the major roads, turning probability *p*_*i*_ that a vehicle turns at the road intersection can be calculated as the weight of the road it turns to divided by the sum of the weights it theoretically can turn. However, it is rare for vehicles to turn round directly at the intersection. Thereby, the probability that a vehicle moves from *o* to *d* by the SP can be calculated by accumulating all the turning probabilities at all the road intersections as shown in Equation 1.

1 $${P}_{o\to d}=\prod _{i=1}^{n}{p}_{i},({p}_{i}=\frac{wg{t}_{turns}}{\sum (wg{t}_{can\_turn})-wg{t}_{has\_traveled}})$$

### Road networks discretization and generalization

Since UHI is a field phenomenon, summarizing heat flux produced by vehicles with enclosed regions is convincing. To maintain the universality, road networks are thus discretizated by a set of homogeneous grid cells that *touch* ^50^ with each other in the same cell resolution *cr*, and each cell *c*(*h*, *v*) is referenced by horizontal and vertical indices starting from the cell *c*(1,1). Certainly, each *c*(*h*, *v*) has four boundaries in the east, west, south and north directions and each boundary *b* is noted as *e*, *w*, *s* and *n*, respectively. In each cell, average length of all the directional SPs that vehicles can move from one boundary to each of the four boundaries is summarized as ${D}_{b\to b^{\prime} }=\frac{\sum lengt{h}_{sp}}{nu{m}_{sp}},(b,b^{\prime} \in \{e,w,s,n\})$. Similarly, average length of the directional SPs that vehicles can travel from one traffic counting station *s*_*p*_ (*p* = 1, …, *q*) to any one of the four boundaries *b*′ can also be generalized and noted as ${D}_{{s}_{p}\to b^{\prime} }$. Notably, SP calculation for both ${D}_{{s}_{p}\to b^{\prime} }$ and *D*_*b*→*b*′_ is only based on the discretized road networks that fall in the single cell. Further, based on the maximum limited speed *S*_°_ for each directional road line segment, the averaged maximum speed for each SP $s{p}_{{s}_{p}\to b^{\prime} }$ and *sp*_*b*→*b*′_ are calculated as ${S}_{\circ }=\frac{\sum maxSp{d}_{line}}{nu{m}_{line}}$, respectively.

### Station-to-boundary and boundary-to-boundary accessibilities

A road may intersect with a boundary as a *node*, and there can be several *nodes* $\{n{d}_{i}^{(h,v,b)},(i=0,\mathrm{...},j)\}$ in the same boundary that vehicles can move to from a counting station. Thus, the station-to-boundary (S-B) accessibility can be represented by the *joint probability* that a vehicle theoretically can move from a traffic counting station *s*_*p*_ to a neighbouring boundary *b*′ by the SPs, and the accessibility can be calculated as the sum of all the SP-based moving probabilities to the same boundary as represented in Equation 2. Simultaneously, a vehicle can also move from *b* to *b*′ in the same cell, and the probability that it moves from *b* to *b*′ similarly can be represented by the boundary-to-boundary (B-B) accessibility and computed in Equation 3. Since the two accessibilities represent the direct accessing abilities of vehicles between S-B and B-B without acrossing any boundaries, both calculations are based on the discretized roads in a single cell. Thus, S-B and B-B dummy paths in each cell are established as $D{P}_{{s}_{p}\to b^{\prime} }=\langle {D}_{{s}_{p}\to b^{\prime} },{A}_{{s}_{p}\to b^{\prime} },{S}_{\circ }\rangle$ and *DP*_*b*→*b*′_ = 〈*D*_*b*→*b*′_, *A*_*b*→*b*′_, *S*_°_〉 for traffic flow estimation in the next stage.

2 $${A}_{{s}_{p}\to b^{\prime} }=\sum _{j=0}^{n}{P}_{{s}_{p}\to n{d}_{j}}$$

3 $${A}_{b\to b^{\prime} }=\sum _{i=0}^{m}\sum _{j=0}^{n}{P}_{n{d}_{i}\to n{d}_{j}}$$

### Elimination of duplicately-counted vehicles

For an easy representation, let a tuple *SW* = (*t*_*wsize*_, *t*_*wstep*_, *t*_*w*_) be a temporal *sliding window* that *t*_*wsize*_ slides forward at each time instant *t*_*w*_ for each *t*_*wstep*_ time period named as the *window step*, such that non-overlapping streaming data can be used for incremental statistics when *t*_*wsize*_ equals to *t*_*wstep*_. Let *T* = *w* ⋅ *t*_*wstep*_ denote an incremental time period for the past *w* window steps. Utilizing traffic counting stations to simulate dynamic vehicular flows is useful for accurately estimating the number of the moving vehicles, which, however, causes a non-ignorable problem that some vehicles may pass through two or several counting stations located in the same statistical cell. This implies that these vehicles will be duplicatedly counted, since all the vehicles that pass through counting stations located in the same cell will be accumulated for each time period *t*_*wsize*_. To eliminate this scenario, a concept of the station-to-station (S-S) accessibility is proposed. For each cell *c*(*h*, *v*) and for each pair of counting stations *s*_*p*_ and *s*_*q*_ located in the same cell, it computes the probability that a vehicle moves from upstream *s*_*p*_ to downstream *s*_*q*_ along the SP. Let ${A}_{{s}_{p}\to {s}_{q}}$ denote the S-S accessibility and equal to the continued multiplication of the turning probabilities when the vehicle turns at the intersections in the SP. Different from the S-B and B-B accessibility calculation, original roads without the cell-clipping are used to calculate the S-S accessibility. Therefore, the generated SP allows a vehicle to cross boundaries of the same cell several times to continuously stay in the original cell during the *t*_*wsize*_. If a number of *n* vehicles pass through *s*_*p*_ at *t*_*w*_, it is reasonable to infer that dispersion of these vehicles follow the multinomial distribution and certain number of the vehicles will travel to *s*_*q*_. The number of the dispersed vehicles generally follows the multinomial distribution as shown in Equation 4 with the *maximum expected value*. When ${A}_{{s}_{p}\to {s}_{q}}$ is obtained, a number of $n\cdot {A}_{{s}_{p}\to {s}_{q}}$ vehicles will arrive at the *s*_*q*_ during the current *t*_*wszie*_ in a high possibility. Thus, the duplicated counting is eliminated when the number of the vehicles from the *s*_*p*_ is adjusted to $n\cdot (1-{A}_{{s}_{p}\to {s}_{q}})$.

4 $$P({X}_{1}={n}_{1},\mathrm{...},{X}_{j}={n}_{j})=\{\begin{array}{l}n!\prod _{i=1}^{j}\frac{{A}_{{s}_{p}\to {s}_{q}}^{{n}_{i}}}{{n}_{i}!},\sum _{i=1}^{j}{n}_{i}=n\wedge {s}_{q}\in \{{s}_{1},\mathrm{...},{s}_{j}\}\\ \mathrm{0,}\,{\rm{otherwise}}\end{array}$$

**Figure 1:**
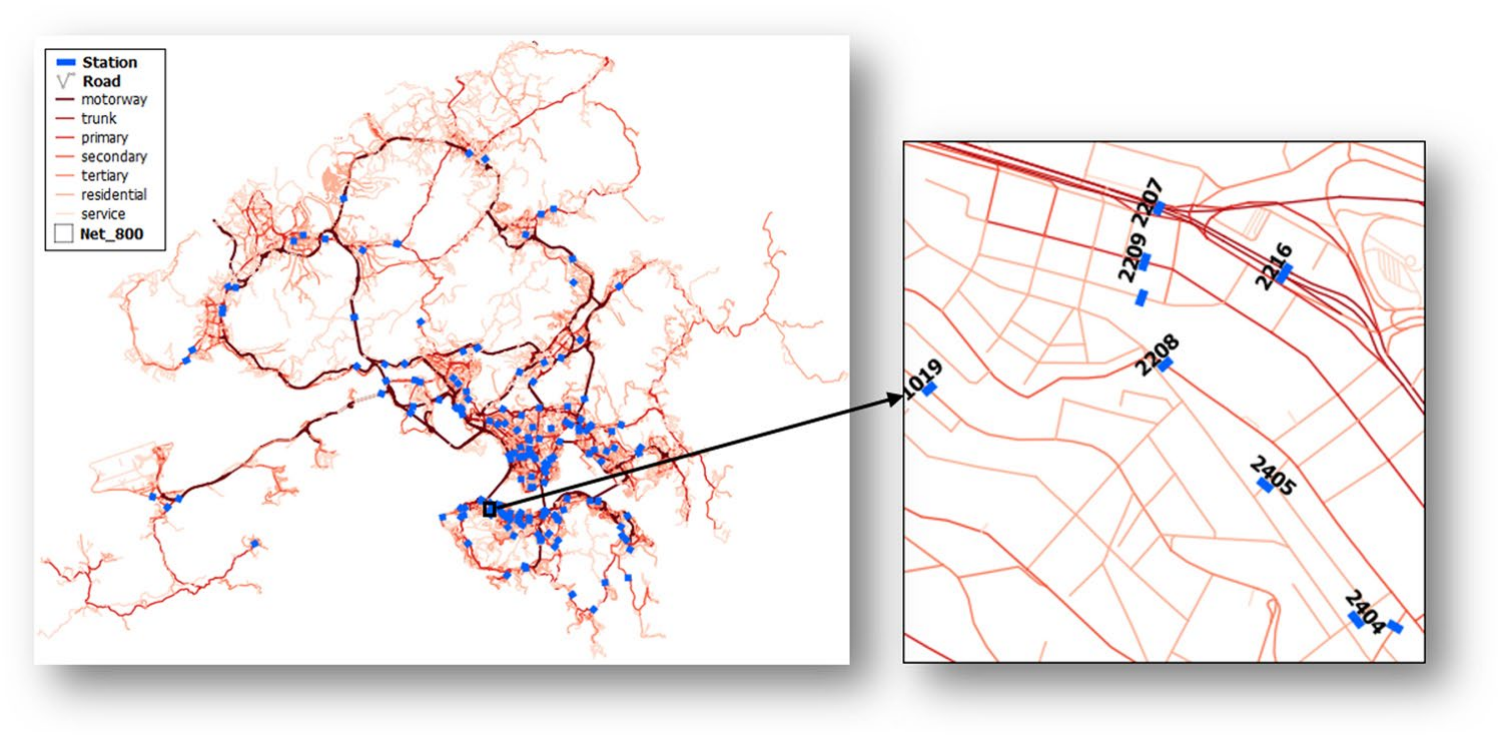
Illustration of the S-S, S-B, and B-B accessibilities. Road networks in Hong Kong are classified into seven types, which are intersected by 169 traffic count stations. Resolution of the discretized grid cell in this example is 800 meters, and the enlarged cell is *c*(41,13), in which there are nine stations and each one accumulates the passing vehicles in one or more lanes. The figure is originally created by using ArcMap 10.0 (URL link: http://support.esri.com/Products/Desktop/arcgis-desktop/arcmap/10), in which the data are in the ESRI shapefile format.

Figure 1 describes an instance that road networks in Hong Kong are discretized by grid cells in 800 meters, and there are 9 traffic counting stations locating in the cell *c*(41,13). Correspondingly, detailed description of the B-B, S-B, and S-S accessibilities for the cell are represented in a table directly plotted from the table in PostgreSQL 9.5 (Table 1). It is suggested that the highest probability for vehicles traveling to the downstream east boundary is from the north boundary. Simultaneously, vehicles traveling forward to the east boundary can also start from five counting stations in theory, in which two stations (their IDs are 2209 and 2402) are more likely to lead the vehicles to the east boundary. Investigating the S-S accessibility, vehicles can move from the 2216 counting station to six other counting stations, and half of the vehicles most probably will move to the 2207 station. Therefore, it is intuitively believed that Table 1 can effectively reveal dummy paths characteristics for each statistical cell.

**Table 1:** Accessibility of vehicles for the dummy paths of boundary-to-boundary, station-to-boundary, and station-to-station.

*X*	*Y*	*b*	*b*′	*A*_*b*→*b*′_	*D*_*b*→*b*′_ (*m*)	*S*_*b*→*b*′_ (*km/h*)
41	13	w	e	0.053712	1147.18	70.00
41	13	n	e	0.927163	907.43	70.39
41	13	e	e	0.584512	787.62	70.00
41	13	s	e	0.045546	1043.20	70.00
***X***	***Y***	***sp***	***b*** **′**	${{\boldsymbol{A}}}_{{{\boldsymbol{s}}}_{{\boldsymbol{p}}}{\boldsymbol{\to }}{{\boldsymbol{b}}}^{{\boldsymbol{^{\prime} }}}}$	${{\boldsymbol{D}}}_{{{\boldsymbol{s}}}_{{\boldsymbol{p}}}{\boldsymbol{\to }}{{\boldsymbol{b}}}^{{\boldsymbol{^{\prime} }}}}{\boldsymbol{(}}{\boldsymbol{m}}{\boldsymbol{)}}$	${{\boldsymbol{S}}}_{{{\boldsymbol{s}}}_{{\boldsymbol{p}}}\to {{\boldsymbol{b}}}^{{\boldsymbol{^{\prime} }}}}$ **(** ***km/h*** **)**
41	13	2207	e	0.500000	526.25	76.00
41	13	2209	e	0.776263	686.77	70.00
41	13	2210	e	0.286170	977.96	70.00
41	13	2216	e	0.500000	308.53	73.33
41	13	2402	e	0.760590	911.37	70.00
***X***	***Y***	***s***_***p***_	***s***_***q***_	${{\boldsymbol{A}}}_{{{\boldsymbol{s}}}_{{\boldsymbol{p}}}{\boldsymbol{\to }}{{\boldsymbol{s}}}_{{\boldsymbol{q}}}}$		
41	13	2216	2207	0.500000		
41	13	2216	2208	0.000133		
41	13	2216	2209	0.001678		
41	13	2216	2402	0.000114		
41	13	2216	2404	0.000092		
41	13	2216	2405	0.000046		

### Station-to-boundary traffic flow simulation

This work assumes that all the vehicles start from traffic counting stations and move in the SPs to approach their destinations, which means origins of all the OD pairs are easily determined as a set of {*s*_*p*_}. To simulate S-B vehicular flows (i.e. ${f}_{{s}_{p}\to b^{\prime} }$), it imports streaming data of $\{{n}_{{s}_{p}}^{w}\}$ into the road networks at each *t*_*w*_, which simulates the process that *n* number of vehicles simultaneously pass through each specific counting station *s*_*p*_ in the bidirectional or unidirectional streets at *t*_*w*_. These vehicles are supposed to move to the four boundaries of the cell where they are located in, and dispersion of these vehicles to the boundaries also follow the multinomial distribution. In this scenario, the number of the vehicles dispersing to each *b*′ equals to ${A}_{{s}_{p}\to b^{\prime} }\cdot {n}_{{s}_{p}}^{w}$ given the assumption that they always follow the *maximum expected value*. To accumulate the total number of the vehicles ${N}_{{s}_{p}\to b^{\prime} }^{w}$ that are traveling from *s*_*p*_ to *b*′ during the current *t*_*wsize*_, the number of vehicles have already arrived at or passed through *b*′ during the past *t*_*wsize*_ have to be determined. This can be achieved by recording the traveled distance information for each set of vehicles during the past (*w* − 1) window steps as $\{({n}_{{s}_{p}\to b^{\prime} }^{i},{d}_{{s}_{p}\to b^{\prime} }^{i})\}$ (*i* = 1, …, *w* − 1), which is updated based on an alternative option setting as shown in Equation 5. Thus, the number of vehicles that have passed through *b*′ during the past *t*_*wsize*_ can be accumulated and noted as ${N}_{{s}_{p}\Rightarrow b^{\prime} }^{w-1}$. Hence, ${N}_{{s}_{p}\to b^{\prime} }^{w}$ can be computed in Equation 6. Based on the ${N}_{{s}_{p}\to b^{\prime} }^{w}$ that has already obtained, the distance that each set of the vehicles can travel from *s*_*p*_ to *b*′ during the current *t*_*wsize*_ is computed as ${l}_{{s}_{p}\to b^{\prime} }=sp{d}_{{s}_{p}\to b^{\prime} }\cdot {t}_{wsize}$ and the traveled distance is updated in each iteration in Equation 7.

5 $${n}_{{s}_{p}\to b^{\prime} }^{i}=\{\begin{array}{cc}{n}_{{s}_{p}\to b^{\prime} }^{i}, & {\rm{i}}{\rm{f}}\,{d}_{{s}_{p}\to b^{\prime} }^{i} < {D}_{{s}_{p}\to b^{\prime} }\\ 0, & {\rm{o}}{\rm{t}}{\rm{h}}{\rm{e}}{\rm{r}}{\rm{w}}{\rm{i}}{\rm{s}}{\rm{e}}\end{array}$$

6 $${N}_{{s}_{p}\to b^{\prime} }^{w}={N}_{{s}_{p}\to b^{\prime} }^{w-1}+{A}_{{s}_{p}\to b^{\prime} }\cdot {n}_{{s}_{p}}^{w}-{N}_{{s}_{p}\Rightarrow b^{\prime} }^{w-1}$$

7 $${d}_{{s}_{p}\to b^{\prime} }^{i}={d}_{{s}_{p}\to b^{\prime} }^{i}+{l}_{{s}_{p}\to b^{\prime} },\forall i\in \mathrm{\{1},\mathrm{...},w\}$$

### Boundary-to-boundary traffic flow simulation

Vehicles which have passed through *b*′ from *s*_*p*_ are in the downstream cell *c*(*h* + *k*, *v* + *l*) ((*k*, *l*) ∈ {(±1, 0), (0, ±1)}), sharing the same boundary *b*′ with the *c*(*h*, *v*). These vehicles are assumed to move continuously from *b*′ to the boundary *b*′′ (*b*′′ ∈ {*e*, *w*, *s*, *n*}) of the downstream cell. To simultaneously simulate B-B vehicular flows (i.e. ${f}_{b^{\prime} \to b^{\prime\prime}}$) for the current *t*_*wsize*_, the number of vehicles that have respectively moved into *b*′ and moved out of *b*′′ during the past *t*_*wsize*_ has been determined firstly. Similar to the approach above, the number of vehicles and travel distances of the vehicles that moved from *b* to *b*′ in each *c*(*h*, *v*) are recorded as $\{({n}_{b{\to }_{b^{\prime} }}^{i},{d}_{b\to b^{\prime} }^{i})\}$ (*i* = 1, …, *w* − 1). Vehicles that move into *c*(*h* + *k*, *v* + *l*) through *b*′ at *t*_*w*_ can come from *s*_*p*_ and *b* in the *c*(*h*, *v*) that satisfy ${d}_{{s}_{p}\to b^{\prime} }^{i}\ge {D}_{{s}_{p}\to b^{\prime} }$ and ${d}_{b\to {b}^{{\rm{^{\prime} }}}}^{i}\ge {D}_{b\to {b}^{{\rm{^{\prime} }}}}$, respectively. Thereby, the total number of these vehicles can be noted as ${n}_{\Rightarrow b^{\prime} }^{w}$ and be aggregated in Equation 8. Assuming all the vehicles from *b*′ to *b*′′ also follows the *maximum expected value* in the multinominal distribution for the B-B accessibility, the newly-entered vehicles disperse to each specific *b*′′ can be determined as ${A}_{b^{\prime} \to b^{\prime\prime}}\cdot {n}_{\Rightarrow b^{\prime} }^{w}$. Simultaneously, the total number of the vehicles that have left *c*(*h* + *k*, *v* + *l*) from *b*′ via *b*′′ during the past *t*_*wsize*_ can be summarized and denoted as ${N}_{b^{\prime\prime} \Rightarrow }^{w-1}$, meeting the condition that ${d}_{b^{\prime} \to b^{\prime\prime} }^{i}\ge {D}_{b^{\prime} \to b^{\prime\prime}}$. Hence, total number of vehicles ${N}_{b^{\prime} \to b^{\prime\prime} }^{w}$ that are moving from *b*′ to *b*′′ in *c*(*h* + *k*, *v* + *l*) during the current *t*_*wsize*_ is updated in Equation 9. Thus, the distance that vehicles can travel from *b*′ to *b*′′ for each *c*(*h* + *k*, *v* + *l*) can be estimated (i.e. *l*_*b*′→*b*′′_ = *spd*_*b*′→*b*′′_ ⋅ *t*_*wsize*_) and total travel distance of all the vehicles that are still in the current cell can be incrementally summarized in Equation 10.

8 $${n}_{\Rightarrow b^{\prime} }^{w}=\sum _{p=1}^{q}{n}_{{s}_{p}\Rightarrow b^{\prime} }^{w-1}+\sum {n}_{b\Rightarrow b^{\prime} }^{w-1},{\rm{\forall }}b\in \{e,w,s,n\}$$

9 $${N}_{b^{\prime} \to b^{\prime\prime} }^{w}={N}_{b^{\prime} \to b^{\prime\prime} }^{w-1}+{A}_{b^{\prime} \to b^{\prime\prime} }\cdot {n}_{\Rightarrow b^{\prime} }^{w}-{N}_{b^{\prime\prime} \Rightarrow }^{w-1}$$

10 $${d}_{b^{\prime} \to b^{\prime\prime} }^{i}={d}_{b^{\prime} \to b^{\prime\prime} }^{i}+{l}_{b^{\prime} \to b^{\prime\prime} },\,\forall i=\{1,\mathrm{...},w\}$$

### Destination estimation

Assuming volume of the vehicles is constant, decreases of number of moving vehicles on the road networks indicate more vehicles in the parking lots. Therefore, temporal distribution of the total number of vehicles parking at the lots is opposite to the number of moving vehicles on the streets. Let *p*_*w*_ denote hourly vehicular-flow percent of counting station *s*_*p*_ at time instant *t*_*w*_, which is equal to the number of vehicles passing through *s*_*p*_ of an hour divided by the total number of vehicles passing through this counting station of a day. To have unique temporal distribution of the parking-vehicles for each specific grid cell containing counting stations, averaged vehicular-flow percent in each *c*(*h*, *v*) is summarized as ${p}_{w}^{ave}$ firstly based on the provided daily statistics so that the daily maximum and minimum values can be determined as *p*_*max*_ and *p*_*min*_. Then, the revised temporal distribution of *p*_*w*_ is computed as ${p}_{w}^{pk}=({p}_{max}+{p}_{min}-{p}_{w}^{ave})$ such that $\{{p}_{w}^{pk}\}$ can represent temporal distribution of vehicles parking at the lots in the corresponding *c*(*h*, *v*). However, an overall temporal distribution of the percent of parking-vehicles is calculated for the grid cells that not containing any counting stations, utilizing the records of vehicular-flow percent of all the counting stations. The number of the parking lots in each cell *c*(*h*, *v*) can be summarized and denoted as $\{{n}_{(h,v)}^{pk}\}$. Assuming vehicles moving in a specific *f*_*b*′→*b*′′_ in the *c*(*h*, *v*) and some of them parking at a set of lots (i.e. ${n}_{(h,v)}^{pk}$) located at the *c*(*h*, *v*) at *t*_*w*_, the number of vehicles parking at each lot follows a homogeneous temporal distribution as what has been established. For each time period *t*_*wsize*_ and for each *f*_*b*′→*b*′′_ of *c*(*h*, *v*) that the vehicles are moving, the number of stopped vehicles can be calculated as ${n}_{w}^{b^{\prime} \nrightarrow b^{\prime\prime} }={n}_{(h,v)}^{pk}\cdot {p}_{w}^{pk}\cdot {n}_{w}^{b^{\prime} \to b^{\prime\prime} }$. Thus, the number of moving vehicles in the *f*_*b*′→*b*′′_ can be updated accordingly.

### Heat emission accumulation

The study simulates the B-B vehicular flows for each *t*_*wsize*_ based on the deterministic OD pairs. Origins of these flows are cells of the locations of traffic counting stations, and destinations are cells with the parking lots. For each cell *c*(*h*, *v*) and for each *T*_*j*_ (*T*_*j*_ = *n* ⋅ *t*_*wsize*_, *where j* ∈ {1, …, *w*/*n*}) time period, it summarizes the total length that all vehicles have traveled in Equation 11. To estimate the accumulation of heat emission of these vehicles, fuel combustion of different types of the vehicles have to be determined since it can cause significant variation of heat emissions. However, traffic counting stations are not able to identify the type of vehicles. Previous work has proposed an effective model to compute hourly heat emission released from the fuel combustion of vehicles^51^. In this regard, our study refines this model as Equations 12 and 13 to calculate heat emission from vehicles by assuming an evenly distribution of different types of vehicles. Equation 12 calculates the energy (*J m* ^−1^) that each vehicle in type *e* can generate when it travels each meter consuming fuel type *f*, and Equation 13 accumulates the energy (*J*) that all the vehicles have generated in *c*(*h*, *v*) for *T*_*j*_. Specific meanings of other variables used in the two equations are represented in Table 2.

11 $$T{L}_{j}^{(h,v)}=\sum _{i=1+(j-\mathrm{1)}n}^{jn}({N}_{b^{\prime} \to b^{\prime\prime} }^{i}\cdot {l}_{b^{\prime} \to b^{\prime\prime} }^{i})\,[m]$$

12 $$E{V}_{ef}=\frac{NH{C}_{f}\cdot {\rho }_{f}}{F{E}_{ef}}[J\,{m}^{-1}]$$

13 $${E}_{j}^{(h,v)}=\sum _{x=1}^{e}\sum _{y=1}^{f}T{L}_{j}^{(h,v)}\cdot f{r}_{y}\cdot e{r}_{x}\cdot E{V}_{ef}\cdot \frac{n\cdot {t}_{wsize}}{3600}[J]$$

**Table 2:** Specific meaning of the variables used in the equations.

Variables	Meaning
*NHC*_*f*_ (*J kg* ^−1^)	Net combustion of fuel type *f*
*ρ*_*f*_ (*kg l* ^−1^)	Fuel density
*FE*_*ef*_ (*m l* ^−1^)	Mean fuel economy for vehicles in type *e* using fuel type *f*
*fr*_*y*_	Ratio of the vehicles consuming fuel type *f*
*er*_*x*_	Ratio of the vehicles in type *e*
$\frac{n\cdot {t}_{wsize}}{3600}(s)$	Time period for each accumulation
$T{L}_{j}^{(h,v)}(m)$	Total length that all the vehicles have traveled in *c*(*h*, *v*) for *Tj*

Vehicles in Hong Kong mainly consume three types of fuels, including petrol, diesel, and liquefied petroleum (LP) gas. Their net heat combustion (*NHC*_*f*_) and the corresponding fuel density (*ρ*_*f*_) are summarized in Table 3. To compute ${E}_{j}^{(h,v)}$, ratio of the fuel type (*fr*) and ratio of the vehicle type (*er*) that uses *fr* are also required, which are computed as combined ratio (*r*) in Table 4 together with the corresponding mean fuel economy (*FE*_*ef*_). Based on the assumption that different types of the vehicles have uniform distribution in Hong Kong respectively, ${E}_{j}^{(h,v)}$ is thus computed when $T{L}_{j}^{(h,v)}$ has been obtained during each *T*_*j*_.

**Table 3:** Net heat combustion and fuel density of fuels.

Fuel type	Net heat combustion (×10^6^ *J kg* ^−1^)	Fuel density (*kg l* ^−1^)
Petrol	46.4	0.75
Diesel	42.8	0.85
LP Gas	50.2	0.58

**Table 4:** Ratio and heat flux for different types of the vehicles.

Vehicle type	Fuel type	Total num	Ratio (%)	Mean fuel economy (*m l* ^−1^)
Motor cycle	Petrol	47710	0.066470	24390.24
Private car	Petrol	512808	0.714450	7800.31
Private car	Diesel	5280	0.007356	9259.26
Taxi	LP Gas	18100	0.025217	6341.15
Single deck bus	Diesel	7687	0.010710	4140.79
Double deck bus	Diesel	5786	0.008061	1592.36
Light bus	Diesel	3612	0.005032	5479.45
Light bus	LP Gas	3795	0.005287	3412.97
Light goods vehicle	Petrol	801	0.001116	8547.01
Light goods vehicle	Diesel	69567	0.096922	7633.59
Medium goods vehicle	Diesel	35999	0.050154	3636.36
Heavy goods vehicle	Diesel	6621	0.009224	2617.80

Total numbers of different types of vehicles are summarized based on the source data from Transport Department of Hong Kong^48^, and the mean fuel economy values of the vehicles are calculated based on the source data from Electrical and Mechanical Services Department of Hong Kong^49^. Principle of the categorization is based on the amount of fuels that vehicles consume.

### Accuracy test

To test the accuracy of simulated vehicular flows, the number of vehicles collected from counting stations are utilized to compare with those from estimation. Since the model has recorded the number of vehicles ${n}_{\Rightarrow b^{\prime} }^{w}$ that enter into a new grid cell from the boundary *b*′ during each *t*_*wsize*_ benefiting from the B-B vehicular flow simulation, these vehicles are supposed to continuously move forward and arrive at the counting stations {*s*_*p*_} in the current grid cell. This can be achieved by calculating the boundary-to-station (B-S) accessibility that, for each grid cell where counting stations are located in, vehicles move from each *b*′ to each *s*_*p*_. Using the same method as proposed, the probability that each vehicle can move from a *node nd*_*j*_ intersected by a road and a boundary *b*′ to a counting station *s*_*p*_ can be calculated and noted as ${P}_{n{d}_{j}\to {s}_{p}}$. Theoretically, vehicles tending to travel on the roads have higher weights, and dispersion of the vehicles on the boundary *b*′ can be determined based on the given weights of the roads. In this consideration, the accessibility that vehicles at *node nd*_*j*_ moving from *b*′ to *s*_*p*_ is calculated as in Equation 14. Ultimately, the number of vehicles moving from *b*′ to *s*_*p*_ is obtained as ${A}_{b^{\prime} \to {s}_{p}}\cdot {n}_{\Rightarrow b^{\prime} }^{w}$ for accuracy assessment.

14 $${A}_{b^{\prime} \to {s}_{p}}=\sum _{j=0}^{n}(\frac{wg{t}_{n{d}_{j}}}{\sum wg{t}_{n{d}_{j}}}{P}_{n{d}_{j}\to {s}_{p}})$$

**Figure 2:**
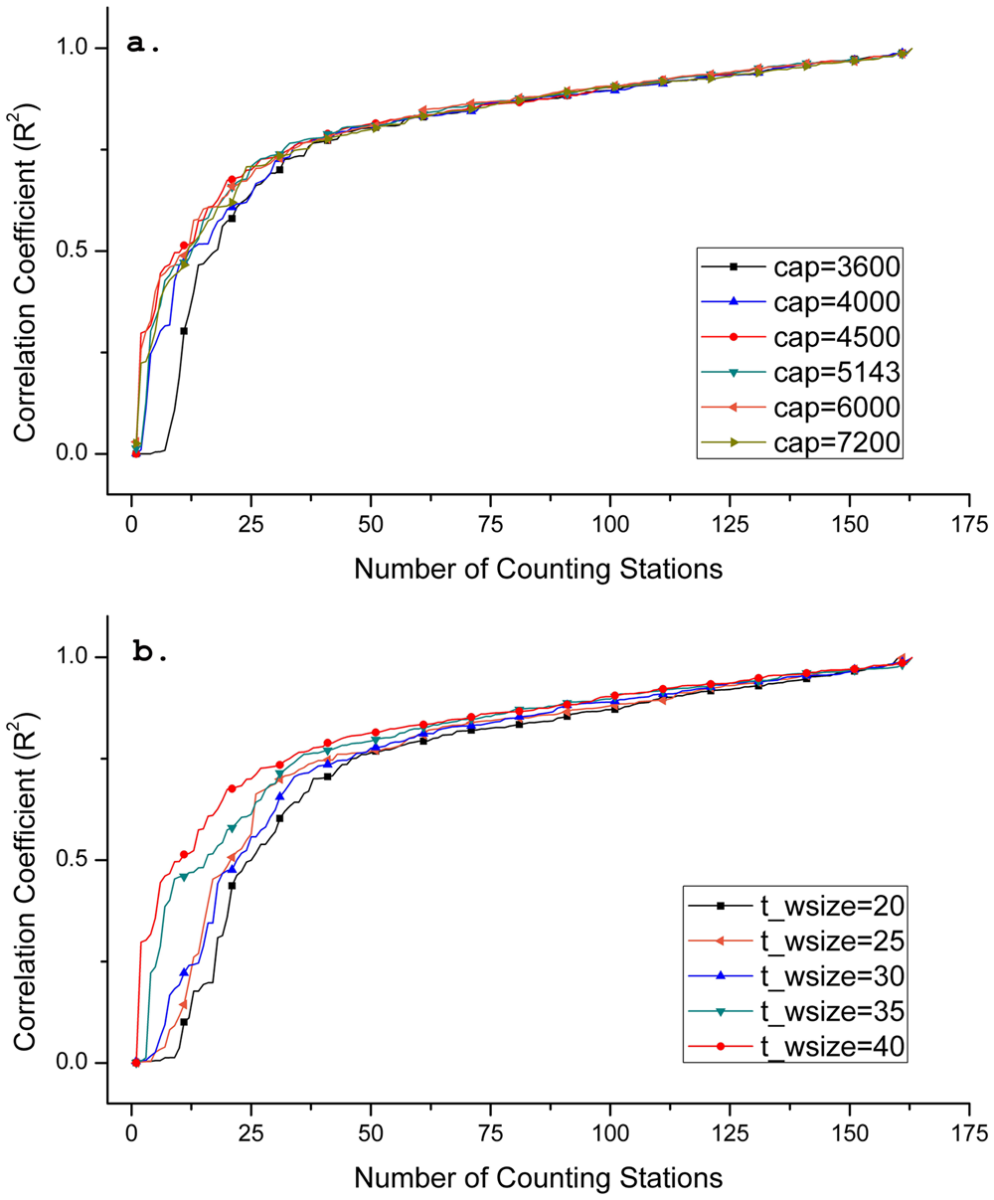
Accuracy test for the vehicular-flow estimation investigating two fundamental parameters when cell resolution is 800 meters. (**a**) Correlation coefficients (*R* ^2^) between measured and estimated vehicles for all the counting stations when the *capacity* is set from 3600 and 7200. (**b**) Correlation coefficients (*R* ^2^) between measured and estimated vehicles for all the counting stations when *t*_*wsize*_ is set from 20 to 40 seconds.

**Figure 3:**
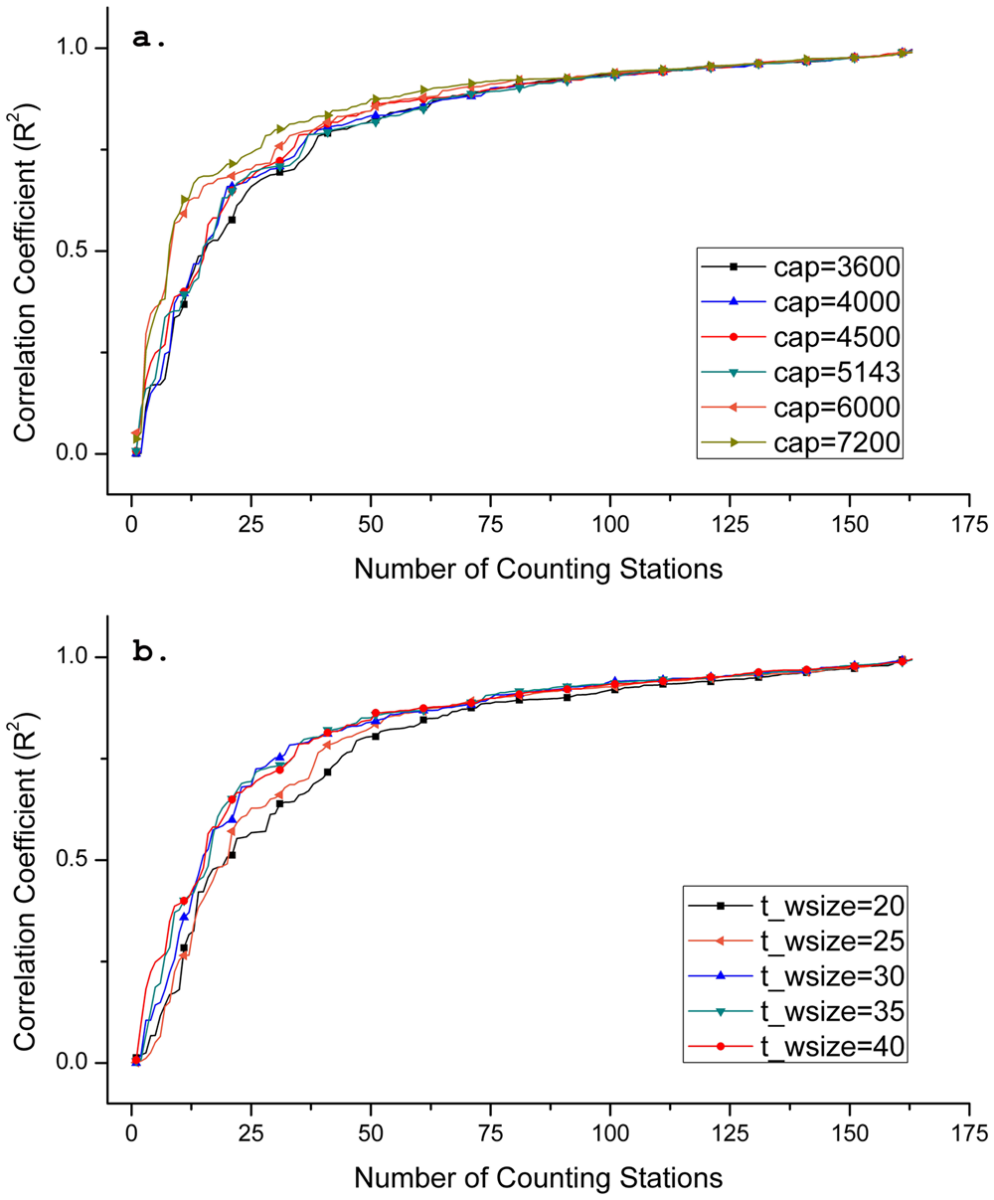
Accuracy test for the vehicular-flow estimation investigating two fundamental parameters when cell resolution is 400 meters. (**a**) Correlation coefficients (*R* ^2^) between measured and estimated vehicles for all the counting stations when the *capacity* is set from 3600 to 7200. (**b**) Correlation coefficients (*R* ^2^) between measured and estimated vehicles for all the counting stations when *t*_*wsize*_ is set from 20 to 40 seconds.

Weights (*wgt*) for different types of roads were determined based on the averaged number of total vehicles passing through the corresponding types of roads, and *wgt* for *motorway*, *trunk*, *major*, *secondary*, *tertiary*, *residential*, and *service* is equal to 31, 39, 14, 6, 7, 3, and 1, respectively. To investigate the influence of different spatial granularities on accuracy of estimation, cell resolutions (*cr*) in 800 and 400 meters were used for two independent testings. For each *cr*, two vital parameters were then investigated, i.e., *capacity* (*cap*) to estimate travel speed and *window size* (*t*_*wsize*_) to determine each simulation time period. Figures 2 and 3 depicts the curves of the correlation coefficients (*R* ^2^) between the observed and the estimated number of vehicles listed in an ascending order in two resolutions, using the data in All-day statistics to significantly reduce the time consumption of the estimation. In particular, when *cap*, *t*_*wsize*_, and *cr* are respectively equal to 4500 vehicles, 40 seconds, and 800 meters, refined algorithms and improved execution program in PostgreSQL can reduce the time cost from the original of more than 5 minutes for 480,000 records to 16~20 seconds for 800,000 records to complete each iterative computation in the database. Time cost does not significantly extend with finer spatio-temporal scales (*cr* = 400 meters and *t*_*wsize*_ = 20 seconds). Assuming that each road at the maximum capacity allows vehicles to pass through the road from 0.5 second per vehicle (i.e. 7200 vehicles per hour) to 1.0 second per vehicle (i.e. 3600 vehicles per hour) with setting *t*_*wsize*_ constantly equaling 40 seconds, Fig. 2(a) shows all curves getting similar accuracy trend, and approximate 85% of the {*R* ^2^} are higher than 0.8. While, the curve with *cap* equaling to 4500 vehicles has slightly better performance. By fixing this *capacity*, Fig. 2(b) presents the curves when *t*_*wsize*_ is ranged from 20 (i.e. 180 iterations per hour) to 40 seconds (i.e. 90 iterations per hour). However, the figure shows that accuracy decreases with an increase of the number of iterative computation. Using same setting of these two parameters, Fig. 3(a) shows better and more stable results with different capacities when *cr* is 400 meters. For better comparison, *cap* was also determined as 4500 vehicles to test the accuracy by verifying *t*_*wsize*_ since this capacity can still achieve a satisfactory result. Coincidentally, Fig. 3(b) suggests that *t*_*wsize*_ equaling to 40 seconds can also achieve a sound result, which even has more high-correlations (25 more stations are higher than 0.9 for *R* ^2^ compared with that of *cr* equaling to 800 meters).

**Figure 4:**
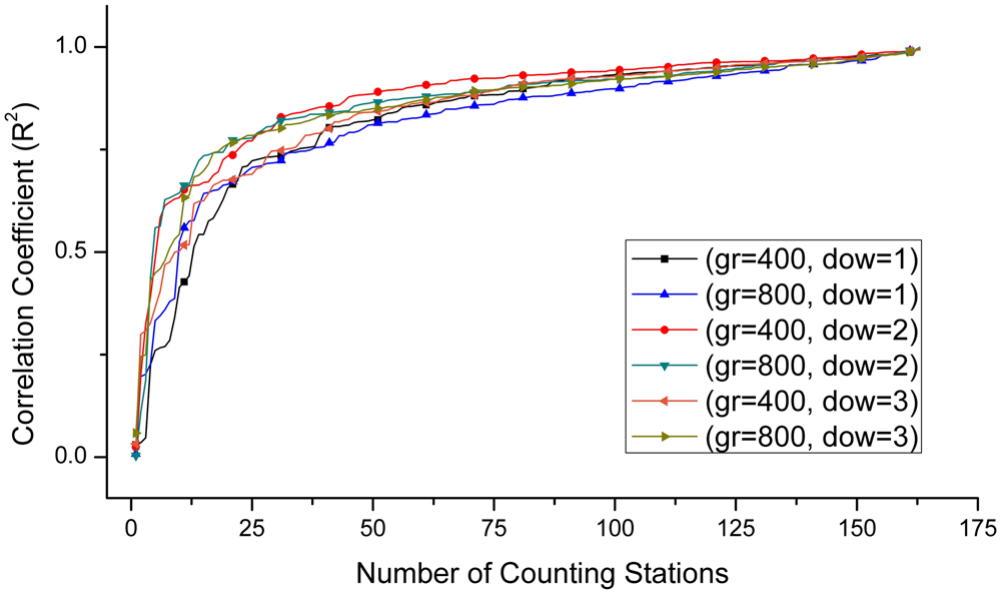
Correlation coefficients (*R* ^2^) between measured and estimated vehicles for all the counting stations and for three continuous days from weekday, Saturday, to Sunday when *t*_*wsize*_ and *cap* respectively equal to 40 seconds and 4500 vehicles.

Based on the results above, 40 seconds of *t*_*wsize*_ and 4500 vehicles of *cap* were used to simulate three continuous days of the vehicular flows from weekday, Saturday, to Sunday. This simulation can also reduce computational cost with less iteration. Figure 4 is a comparison of two sets of correlations for all counting stations in three corresponding days derived from 800 and 400 meters of the statistical grid cells. It suggests that both have optimized to high correlations, and all curves grow dramatically to 0.6 at 20 from the very beginning, reach 0.8 at 50, resulting to higher correlations for the rest stations. Particularly for *cr* equaling to 400 meters, weekday (*dow* = 1) and Saturday (*dow* = 2) have slightly higher correlations than that of *cr* equaling to 800 meters when {*R* ^2^} are respectively higher than 0.7 and 0.8. While Sunday (*dow* = 3) for *cr* equaling to 400 meters generally has lower correlations for the first 45 stations, and it reaches almost the same correlations for the rest compared with that of *cr* equaling to 800 meters. Thus, heat emission from the estimated vehicles can be accumulated based on two statistical cell resolutions since both can achieve reliable results.

**Figure 5:**
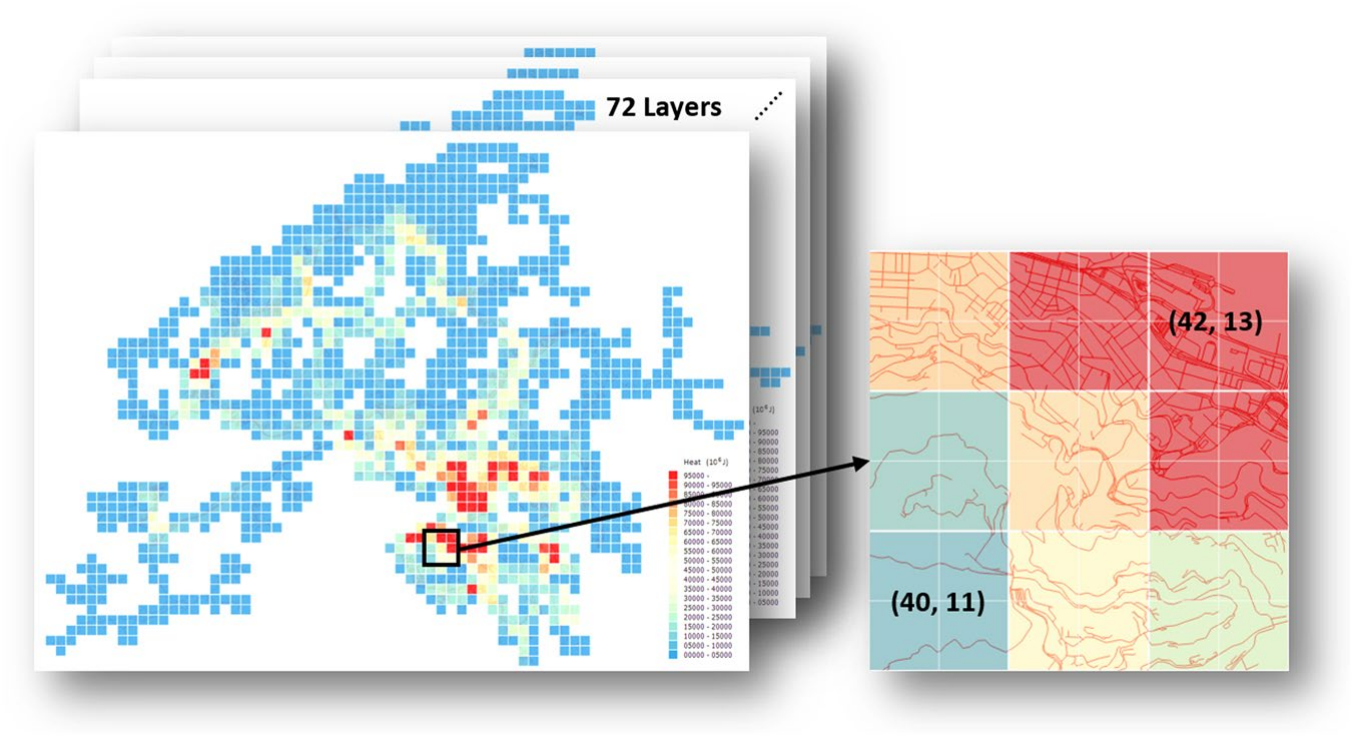
Heat emission from vehicles is accumulated for each grid cell and for each every hour for the three continues days in two different cell resolutions. Spatial continuity of nine grid cells (*cr* = 800 m) between *c*(40, 11) and *c*(42, 13) contain 36 smaller grid cells (*cr* = 400 m) so that heat of each every four smaller grid cells located in the larger one are summarized for the comparison of that of the larger grid cell. QGIS Desktop 2.8.2 (http://www.qgis.org/en/site/) is used to connect the DBMS PostgreSQL 9.5 (https://www.postgresql.org/) so that the 72 layers stored in the database can be read and visualized in sequence to have a screenshot to create the figure in Adobe Photoshop 7.0 (http://www.adobe.com/products/photoshopfamily.html).

**Figure 6:**
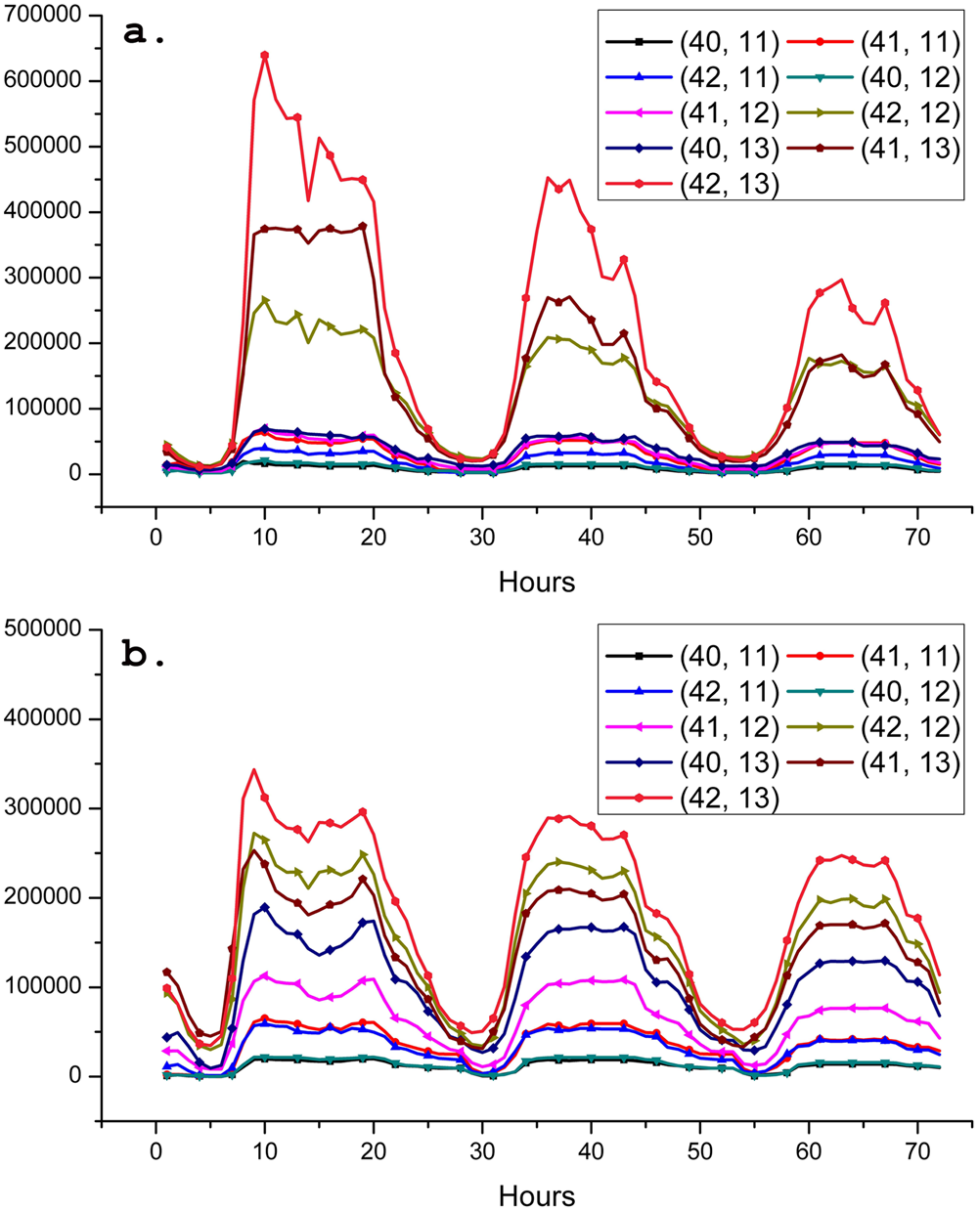
Comparison of the estimated heat summarized by nine spatial continuity of grid cells (*cr* = 800 m) from *c*(40, 11) to *c*(42, 13) for continues of weekday, Saturday, and Sunday. (**a**) Hourly accumulated heat for nine spatial continuity of grid cells when *cr* is 800 meters. (**b**) Hourly accumulated heat for each very four spatial continuity of grid cells (*cr* = 400 m) located in the same region of nine larger grid cells.

As shown in Fig. 5, the study accumulated hourly heat generated by vehicles for all grid cells that contain road networks for 72 continuous hours from weekday, to Saturday, and to Sunday. The accumulation was respectively based on *cr* equaling to 800 and 400 meters such that the two sets of the grid cells are spatially coincided with each other. Figure 6 depicts the heat variations for nine enlarged grid cells as represented in Fig. 5. Several distinctive phenomena are found from Fig. 6. First, the total amount of heat generated by vehicles continuously decreases from weekday, Saturday, to Sunday. Second, heat accumulates dramatically since 7 am, reaches the peak at 9 am, and continuously decreases to a valley point at 2 pm, followed by the second peak at 7 pm during the weekday. However, this phenomenon is not significant on Saturday and Sunday. Third, beginning time of rapid heat accumulation in the morning is postponed from 7 to 9 am and the slopes turn to be more gentle from weekday to Sunday. Fourth, the minimum amount of heat occurs during the Sunday night and the early morning of Monday. All of these indicate that periodical human activities in work-and-off days in mega cities such as Hong Kong can influence the micro-environment in the urban areas considerably. i.e., volume of the heat varies through time following the spatio-temporal distribution of moving vehicles. Comparing Fig. 6(a) and (b), the total amount of heat for nine grid cells in each day are almost identical. However, when investigating at each individual grid cell, heat in the *c*(42, 13) and *c*(41, 13) shall be overestimated while heat in the *c*(40, 11) and *c*(40, 13) are supposed to be underestimated in the 800 m grid cells compared with that when *cr* equals 400 m. The reason is that area of the grid cell in 800 m is four times larger than that in 400 m, such that more numbers of vehicles are alternatively determined as staying in the current cell or arriving at a new cell for each iterative vehicular flow estimation. Thus, it is possible to cause over estimation of moving vehicles allocating into current grid cell or its surrounding grid cells. This analysis also suggests that heat summarized by 400 m grid cells has better quantitative coherence in spatial context.

**Figure 7:**
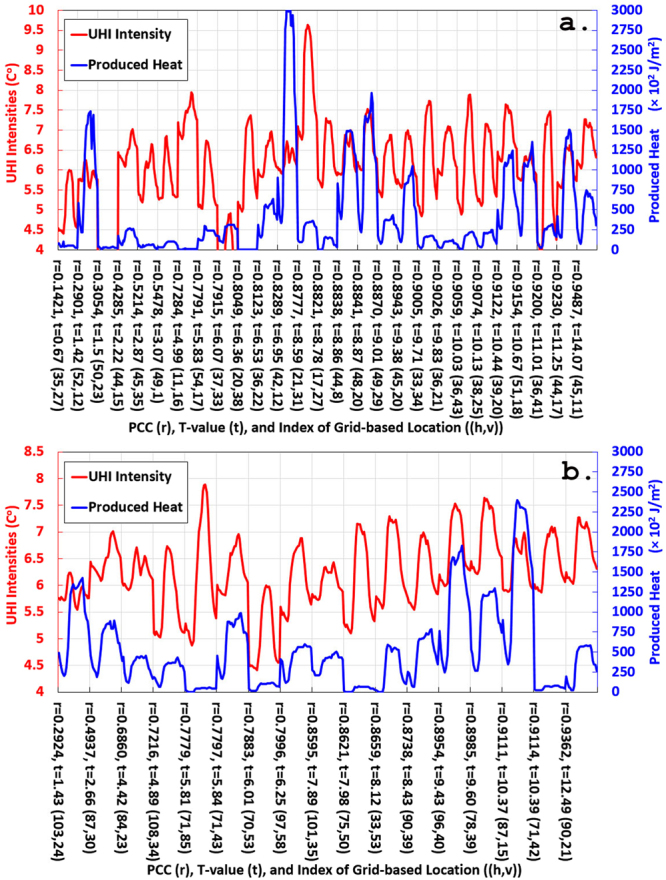
Time series based correlation analysis between the heat produced by vehicles and the measured UHI intensities that are located in the same grid cell. (**a**) Correlation between the estimated heat and the UHI intensities when *cr* is 800 meters. (**b**) Correlation between the estimated heat and the UHI intensities when *cr* is 400 meters.

Air temperature measured by a weather station located in rural area of the New Territories of Hong Kong was used as the reference of rural temperature, so that relative temperatures (i.e. air temperatures measured by other automatic weather stations minus the rural temperature) can be represented as the UHI intensity^52^. Pearson correlation coefficient (PCC denoted as *r*) is used to present the linear correlation between the UHI intensity and the generated heat in the same statistical grid cell. T-test (*p* < 0.01) is also used to explore the significance of correlation. Results show strong positive correlations (when *r* ≥ 0.7) with high confidence (when *t* ≥ 2.819) for the two cell resolutions, i.e., 14 out of 17 (*cr* = 400 m) and 23 out of 27 (*cr* = 800 m) statistical grid cells satisfy this condition. More specifically, Fig. 7 presents hourly UHI intensity of each grid cell, representing locations of weather stations and road networks, averaged heat in the corresponding grid cells, and their *r* and *t* values with the grid-based locations in the horizontal axes.

**Figure 8:**
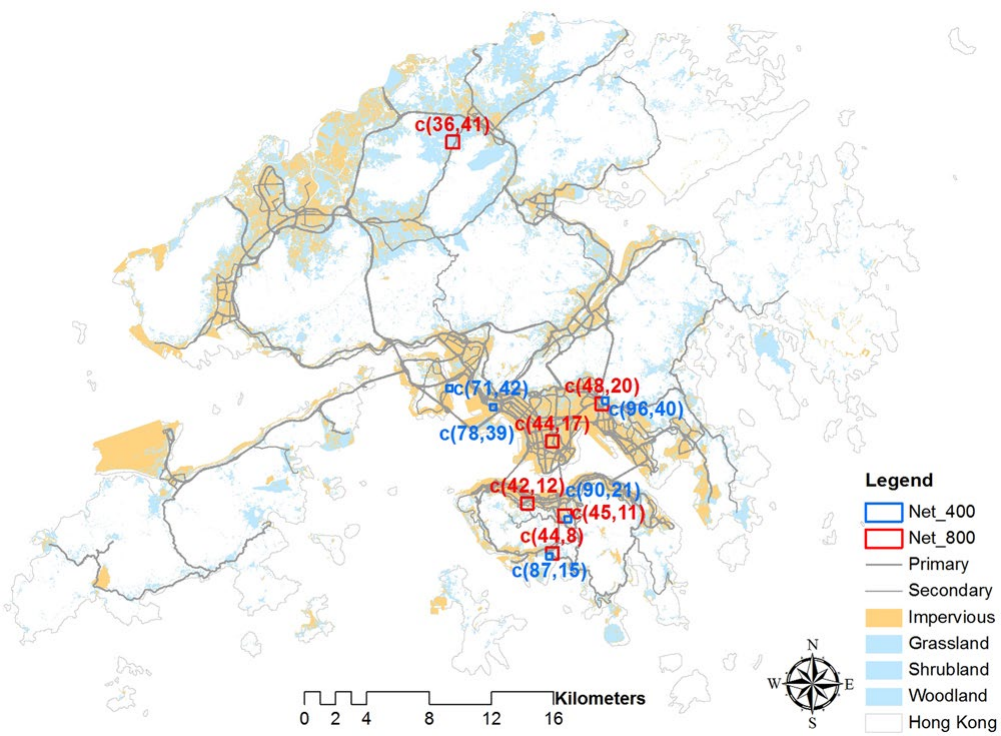
UHI intensities and the heat produced by vehicles are highly correlated in the statistical grid cells, where resolutions of the red and blue cells are 800 m and 400 m respectively. The figure is created in ArcMap 10.0 (http://support.esri.com/Products/Desktop/arcgis-desktop/arcmap/10) with the ESRI shapefile data format.

As shown in Fig. 8, heavy traffic with slow movements or even congestion frequently occurring in the grid cells *c*(42, 12) and *c*(44, 17) should be the main or dominant cause of UHI, because these heavy traffic not only produced the maximum/huge volume of heat but also had strong correlations with UHI intensities (Fig. 7(a)). High-rise buildings surrounded by the roads in the two grid cells are supposed to increase the severity of UHI. Similarly, traffic hubs which a large number of vehicles passing through *c*(48, 20) (a green-land and impervious mixed area) and *c*(78, 39) (an impervious open area) can also be one of the main causative factors since the same factors can be derived in Fig. 7(a) and (b). In contrast, moving vehicles in *c*(71, 42) (Fig. 7(b)), as the only major heat resource in the seaside, had very strong and reliable correlation with the UHI intensities. Even though they produced very small amount of heat, UHI intensities reached as high as 7 degree Celsius. The same phenomenon is also found in the green area (*c*(36, 41) and *c*(45, 11) in Fig. 7(a), and *c*(90, 21) in Fig. 7(b)) where there are less buildings and vehicles are considered to be the only major heat resource. In these two cells, strong and reliable correlations were determined and the heat was insignificant, while intensities still reached up to 7.5 degree Celsius. A similar but different situation occurred in *c*(44, 8) in Fig. 7(a) and *c*(87, 15) in Fig. 7(b) that vehicles accumulated notable amount of heat in the seaside and influenced UHI phenomenon obviously, which is the same as the grid cell *c*(96, 40) (Fig. 7(b)) across green area.

## Discussion

In order to investigate the influence of moving vehicles on UHI phenomenon across urban areas, this study designed a cell-transmission-model to simulate cell-based vehicular flows and to estimate the accumulation of the heat generated from vehicles over continuous time. The study has three important findings. First, heat patterns produced by vehicular flows strictly follow the spatio-temporal patterns of moving vehicles, e.g., heat has peaks and valleys periodically on a daily basis and it also has periodical circulations on a weekly basis. Second, UHI intensities in some specific land covers, such as core area of downtown with a large number of vehicles passing by, and even the green area in the suburbans and seaside with lack of man-made structures, are mainly or dominantly caused by vehicular flows. In other words, vehicular flow is also a considerable influential factor to anthropogenic heat or UHI phenomenon that cannot be ignored. Third, slow movement of vehicles would produce large amount of the heat, which suggests that construction of urban infrastructures (e.g. flyovers and highways) would promote high-speed traffics and hence mitigate the UHI phenomenon effectively for heavily-congested cities. At the same time, if the estimated heat can be precisely transferred as the changed temperature by considering several interactive factors (i.e., atmospheric pressure, heat capacity of the air, and density of air mass under different air temperature in aerodynamics), then the changed temperature can have straight-forward comparison with UHI intensities.

This study designed a unique map generalization method to create dummy paths for each grid cell, so accurate locations of vehicles in the cell is not required, resulting in a significant improvement of effective simulation to fulfill the needs of heat accumulations, i.e., handling with more moving objects over longer time period, but with less time cost, less computational cost and hard disk storage for the intermediate results. This study also solves a fundamental problem of spatio-temporal modeling in transportation by estimating cell-based trajectories of vehicles. In this model, the OD matrix is deterministic because the origins are traffic counting stations and the destinations are grid cells with parking lots and/or roadside parking areas. The OD demand is dynamic since each vehicular flow with the quantitative information varies depending on time. Thus, the model can also be used for other studies. For example, it can be used to simulate patterns of human mobility with a given population census in a detailed spatial scale, and hence to estimate the accumulation of metabolic heat emission of human bodies quantitatively. This metabolic heat emission is also an influential factor of anthropogenic heat that has been widely acknowledged in literature.

## Methods

### Traffic counting data

The Transport Department of Hong Kong provides annual traffic census (ATC) data, which depict up-to-date vehicular flow information of Hong Kong in the year of 2015. Two items of ATC data were used in this study, i.e., geographic information with locations of 169 counting stations that may cross one or more lanes of roads, and textual information including (i) annual average daily traffic (AATD) describing the total number of vehicles passing through the counting stations in the whole year summarized by All-Day, weekday (Monday to Friday), Saturday, and Sunday; and (ii) hourly percentage of the number of vehicles in 24 hours that are also summarized in aforementioned four categories. Thus, the number of vehicles that pass through each station in each hour can be calculated by multiplying two items of the textual information.

### Hourly air temperature

Hourly air temperature collected by 49 automatic weather stations for the whole year of 2015 were acquired from the Hong Kong Observatory. All weather stations were set up around 1–2 meters above the ground in both urban and rural areas. Therefore, air temperature can be an effective indicator to examine the effect of heat emission of vehicular flows since the weather stations and the vehicles are almost at the same heights.

### Road network

It is necessary to determine different types of road network in Hong Kong to assign weight values of the roads for moving probability calculation. For this purpose, this work obtained directional roads from OpenStreetMap, which is an open-source map including volunteered information of road properties. To improve computing efficiency, irrelevant roads such as walking paths were removed and roads having the following seven properties are retained, i.e., *motorway*, *trunk*, *major*, *secondary*, *territory*, *residential*, and *services*.

### Shortest path calculation

Since estimating vehicular flows covering the whole of Hong Kong in a high spatio-temporal resolution is a typical Big Data analytic task, road network and ATC data were thus imported into the PostgreSQL 9.5, an open-source object-relational database management system, to calculate the shortest path and to optimized execution efficiency at the maximum capability. The Dijkstra’s algorithm was used to calculate the shortest path by pgRouting (an extension of PostgreSQL) to provide geospatial routing functionality. To release the computer RAM occupation that can extremely extend the time cost during each iterative computation for the four accessibilities simulation cell after cell and the vehicular flow estimation hour by hour, the central core is built in Eclipses to call the SQL functions and manages all the processes.

### Travel speed estimation

Average speeds of the traffic flows in S-S, S-B, and B-B for each *c*(*h*, *v*) are computed based on an established BPR function^53^ as shown in Equation 15, where *spd*, *S*_°_, *v*, and *c* respectively indicate the average speed in the current traffic condition (i.e. *average link speed*), the maximum speed of the free traffic-flow (i.e. *free-flow link speed*), the number of vehicles that are moving in the streets at the moment (i.e. *volume*), and the number of vehicles that can move at capacity (i.e. *capacity*). The coefficient *a* determines the ratio of the travel time in the free-flow to the travel time at capacity, and *b* determines how rapidly travel time increases starting from the free-flow travel time. The higher the *b* is the less sensitive the estimated travel time will be until the ratio (*v* */* *c*) approaches 1. *a* and *b* are widely set as *0.15* and *4* respectively, and the maximum capacity ratio *v*/*c* is normally between 0.8 and 1.

15 $$spd=\frac{{S}_{\circ }}{[1+a{(\frac{v}{c})}^{b}]}$$
